# Fitness costs of Tn*1546*-type transposons harboring the *vanA* operon by plasmid type and structural diversity in *Enterococcus faecium*

**DOI:** 10.1186/s12941-024-00722-2

**Published:** 2024-07-08

**Authors:** Dokyun Kim, Da Young Kang, Min Hyuk Choi, Jun Sung Hong, Hyun Soo Kim, Young Ree Kim, Young Ah Kim, Young Uh, Kyeong Seob Shin, Jeong Hwan Shin, Soo Hyun Kim, Jong Hee Shin, Seok Hoon Jeong

**Affiliations:** 1 Department of Laboratory Medicine and Research Institute of Bacterial Resistance, Gangnam Severance Hospita, l, Yonsei University College of Medicine, 211 Eonju-Ro, Gangnam-Gu, Seoul, 06273 South Korea; 2 Department of Companion Animal Health and Science, Silla University, Busan, South Korea; 3 Department of Laboratory Medicine, Hallym University Dongtan Sacred Heart Hospital, Hallym University College of Medicine, Hwaseong, South Korea; 4 Department of Laboratory Medicine, Jeju National University College of Medicine, Jeju, South Korea; 5 Department of Laboratory Medicine, National Health Insurance Service, Ilsan Hospital, Goyang, South Korea; 6 Department of Laboratory Medicine, Yonsei University Wonju College of Medicine, Wonju, South Korea; 7 Department of Laboratory Medicine, Chungbuk National University College of Medicine, Cheongju, South Korea; 8 Department of Laboratory Medicine and Paik Institute for Clinical Research, Inje University College of Medicine, Busan, South Korea; 9 Department of Laboratory Medicine, Chonnam National University Medical School, Gwangju, South Korea

**Keywords:** *Enterococcus faecium*, Fitness cost, Tn*1546*, Vancomycin, Teicoplanin

## Abstract

**Background:**

This study analyzed the genetic traits and fitness costs of vancomycin-resistant *Enterococcus faecium* (VREfm) blood isolates carrying Tn*1546*-type transposons harboring the *vanA* operon.

**Methods:**

All *E. faecium* blood isolates were collected from eight general hospitals in South Korea during one-year study period. Antimicrobial susceptibility testing and *vanA* and *vanB* PCR were performed. Growth rates of *E. faecium* isolates were determined. The *vanA*-positive isolates were subjected to whole genome sequencing and conjugation experiments.

**Results:**

Among 308 *E. faecium* isolates, 132 (42.9%) were positive for *vanA*. All Tn*1546*-type transposons harboring the *vanA* operon located on the plasmids, but on the chromosome in seven isolates. The plasmids harboring the *vanA* operon were grouped into four types; two types of circular, nonconjugative plasmids (Type A, n = 50; Type B, n = 46), and two types of putative linear, conjugative plasmids (Type C, n = 16; Type D, n = 5). Growth rates of *vanA*-positive *E. faecium* isolates were significantly lower than those of *vanA*-negative isolates (P < 0.001), and reduction in growth rate under vancomycin pressure was significantly larger in isolates harboring putative linear plasmids than in those harboring circular plasmids (P = 0.020).

**Conclusions:**

The possession of *vanA* operon was costly to bacterial hosts in antimicrobial-free environment, which provide evidence for the importance of reducing vancomycin pressure for prevention of VREfm dissemination. Fitness burden to bacterial hosts was varied by type and size of the *vanA* operon-harboring plasmid.

**Supplementary Information:**

The online version contains supplementary material available at 10.1186/s12941-024-00722-2.

## Background

Enterococci have emerged as one of the leading causes of hospital-associated bacterial infections due to both intrinsic and acquired resistance against many groups of antimicrobials and tolerance to stresses such as disinfectants. *Enterococcus faecalis* caused three-fourths of cases of enterococcal infection in humans in the late 1990s [1]. However, it has repeatedly been reported in recent years that *Enterococcus faecium* exceeds *E. faecalis* in prevalence of human infections, which might be due to rapid adaptation of *E. faecium* to nosocomial conditions by acquiring resistance against anti-enterococcal antimicrobials, including ampicillin, high-level aminoglycosides, and glycopeptides [2]. Furthermore, resistance to glycopeptides in *E. faecium* has been shown to be an important risk factor for an increased early mortality rate and prolonged hospital stays in patients with bloodstream infections [3, 4].

The most common mechanism of resistance against glycopeptides in *E. faecium* is acquisition of an operon harboring *van* genes. Among the nine *van* genotypes, *vanA* (80–90%) and *vanB* (10–20%) have been predominantly identified in vancomycin-resistant *E. faecium* (VREfm), though the proportion of *vanA* and *vanB* genes varies geographically [5, 6]. The *vanA* operon is composed of regulatory genes (*vanR* and *vanS*), genes for peptidoglycan modification enzymes (*vanH*, *vanA*, and *vanX*), and accessory genes (*vanY* and *vanZ*) as a part of Tn*1546*-type transposons located on plasmids [7]. The *vanB* operon includes genes homologous with the *vanA* operon, except that the *vanW* gene instead of *vanZ* is present [8], and it has been commonly identified in Tn*1549* or Tn*5382* on the bacterial chromosome [9, 10].

VREfm of sequence type 17 (ST17) harboring a plasmid with resistance determinant against glycopeptide (VR-plasmid) carrying the *vanA* operon, has been identified as a major clone showing global dissemination; however, regional distribution of VREfm among diverse STs by country or region and shifts in predominating clones over time by emerging successful clones have also been identified. In Australia, exchange of the dominant *vanB*-ST796 VREfm clone by *vanA*-ST1421 has been observed since 2016 [11, 12]. In Germany, rapid dissemination of the *vanB*-ST117 VREfm clone resulted in an inversion in prevalence between *vanA* and *vanB* (from 2:1 to 1:3) in 2019 [13]. In South Korea, *vanA*-ST17 was the predominating VREfm clone since its emergence in the 1990s, but dissemination of emerging *vanA*-ST1421 has recently been reported [14].

Shifts in clonal distribution of VREfm might be affected by multiple factors, including environmental factors such as infection control strategies, antimicrobial pressure, and microbial factors including fitness costs of resistance determinants to bacterial hosts [15, 16]. The fitness cost of acquisition of a plasmid may vary according to the size and replicon type, number and mechanism of resistant alleles, and traits of bacterial hosts [15]; interactions between a plasmid and a bacterial host may also play an important role in determining the cost [16]. In general, possession of a plasmid with resistance determinant may be costly, though it could be nearly cost-free or even beneficial to the bacterial host in some cases [17, 18]. Although fitness costs may be an important factor for emerging successful multidrug-resistant clones, studies on epidemic clones of VREfm are still scarce.

This study was performed to determine the genetic traits of successful VREfm clones in South Korea and their plasmids harboring the *vanA* operon. Determination of the growth dynamics of VREfm blood isolates was also performed to measure the fitness costs of *vanA* operon-containing plasmids for bacterial hosts, which might have an effect on shifts in the clonal distribution of VREfm strains.

## Methods

### Study design

All patients with *E. faecium* bloodstream infection (BSI) between January and December 2019 in eight general hospitals participating in the Global Antimicrobial Surveillance System in South Korea, Kor-GLASS, were included in this study [2]. Clinical information, including demographic conditions, underlying comorbidities, and antimicrobial treatment regimens, was investigated. Hospital-originated infection was defined when an initial blood culture was performed after ≥ 2 calendar days of hospitalization. The Charlson comorbidity index and Sepsis-related Organ Failure Assessment (SOFA) score were calculated as previously described [19, 20]. Clinical outcomes included 30-day mortality, 60-day mortality, and in-hospital mortality. The first *E. faecium* blood isolate from each patient was collected for microbiological studies, and duplicate isolates were discarded.

### Microbiological assessment

Bacterial species were identified using a Bruker Biotyper (Bruker Daltonics, Bremen, Germany) and confirmed by 16S rRNA sequencing. Antimicrobial susceptibility against ampicillin, ciprofloxacin, tetracycline, and quinupristin/dalfopristin was determined by the disk diffusion method. The minimum inhibitory concentrations (MICs) of vancomycin, teicoplanin, linezolid, gentamicin, and streptomycin were determined using the broth microdilution method. Interpretation of zone diameter and MICs were followed the clinical breakpoints of CLSI guideline [21]. *vanA* and *vanB* gene carriage was evaluated by PCR for all *E. faecium* isolates [22, 23].

### Whole-genome sequencing

Genomic DNA was extracted from 132 *vanA*-positive *E. faecium* isolates with a GenElute bacterial genomic DNA kit (Sigma‒Aldrich, St. Louis, MO). Libraries were prepared using SMRTbell Express Template Prep Kit 2.0 (Pacific Biosciences of California, Menlo Park, CA). Entire genomes were sequenced using SMRT cell 1 M by the PacBio Sequel II system (Pacific Biosciences of California). Genome assemblies were performed using PBMM2 (https://github.com/PacificBiosciences/pbmm2; last updated in June 2021), and annotation of the assembled contigs was performed using PROKKA [24].

### Strain typing and phylogenetic analysis

Multilocus sequence typing (MLST) was performed by determining the allelic types of seven housekeeping genes, as previously described [25]. Core genome MLST (cgMLST) was determined by analyzing 1423 loci with the *E. faecium* cgMLST scheme v.1.1 using SeqSphere v.9.0.1 (Ridom GmbH, Munster, Germany) [26]. A cgMLST-based minimum spanning tree was generated with genomic sequences covering > 90% of target loci.

### In silico molecular analysis

Antimicrobial resistance determinants were identified by ResFinder (https://cge.food.dtu.dk/services/ResFinder/) [27], and replicons of plasmids were identified by PlasmidFinder (https://cge.food.dtu.dk/services/PlasmidFinder/) [28]. The bacterial type II toxin-antitoxin system was assessed by comparison with the TADB 2.0 database [29]. NCBI Basic Local Alignment Search Tool was used to compare the structure of plasmids, and plasmid maps were generated using the Proksee online tool (https://proksee.ca/).

### Conjugation

Broth mating was performed to estimate the plasmid transfer frequency of *vanA* operon-harboring plasmids in *E. faecium* isolates using *E. faecium* DSM13589 as a recipient. Mixtures of equal amounts of donor and recipient bacterial cells were incubated in Mueller–Hinton (MH) broth (Difco Laboratories, Detroit, MI) and spread on MH agar (Difco Laboratories) containing fusidic acid (20 mg/L), rifampicin (30 mg/L), and vancomycin (4 mg/L). Putative transconjugant cells were confirmed by antimicrobial susceptibility phenotype and possession of *vanA*, and the conjugation efficiency was calculated per both the number of donor cells and recipient cells.

### Bacterial growth rate

Growth rates of the *E. faecium* isolates were determined by measuring optical density at 600 nm (OD_600_) using a Multiscan spectrophotometer (Thermo Fisher, Waltham, MA). Bacterial colonies were incubated overnight in Luria–Bertani broth (Difco Laboratories) at 37 °C with shaking, and diluted bacterial suspensions were incubated in MH broth while measuring OD_600_ every 3 min. Growth rates of VREfm isolates under both 4 mg/L and 16 mg/L concentrations of vancomycin in MH broth were also determined. Each measurement was replicated three times in the same run, and three independent runs were performed. The average of the maximum slope values of *ln*OD_600_ over time was calculated as the growth rate of the bacterial isolates.

### Statistical analysis

Statistical analyses were performed using R software version 4.3.1 (R development Core Team 2023; http://www.R-project.org/), and the results with *P* value < 0.05 were considered to be significant. Differences between two groups were analyzed using the Mann‒Whitney U test and Fisher’s exact test for continuous variables and categorical variables, respectively. Kruskal–Wallis tests were conducted to determine differences among more than three groups, and significant results were further analyzed using post hoc Dunn’s tests with Bonferroni correction for pairwise comparisons to identify specific groups with significant differences. A Kaplan‒Meier curve was constructed, and the log-rank test was performed. Packages ‘ggpubr’ and ‘ggsurvplot’ were used for visualization of the statistical analysis results.

## Results

### Characteristics of the patients with *E. faecium* BSI

During the one-year study period, blood culture was performed for 87,399 patients with suspected BSI in eight sentinel hospitals, and 10,990 (12.6%) were positive for at least one bacterial or fungal pathogen. Among them, 308 cases (2.8% of positive blood culture) of *E. faecium* BSI were identified and included in this study (Table 1). The median age of the patients was 72.5 years, ranging from 61 to 80 years, and more than half (54.9%, 169/308) were male. Most patients were inpatients in general wards (51.6%, n = 159) and intensive care units (37.0%, n = 114); only 11.4% (n = 35) of them were outpatients. Almost four-fifths (77.9%, n = 240) of cases were hospital-originated infections. The most common underlying comorbidities were malignancies (28.2%, n = 87), followed by diabetes mellitus (16.9%, n = 52) and cardiovascular diseases (16.2%, n = 50).

**Table 1:** Characteristics of the patients with BSI and causative *E. faecium* pathogens

Variables	Total (n = 308)	*vanA*-positive (n = 132)	*vanA*-negative (n = 176)	*P* value
Patient				
Age	72.5 [61.0–80.0]	73.0 [62.0–80.0]	72.0 [60.5–80.0]	0.571
Male	169 (54.9)	71 (53.8)	98 (55.7)	0.830
Inpatient	276 (89.6)	126 (95.5)	150 (85.2)	0.006
Hospital-originated infection	240 (77.9)	119 (90.2)	121 (68.8)	< 0.001
Admission				
Outpatient	35 (11.4)	6 (4.5)	29 (16.5)	< 0.001
General ward	159 (51.6)	66 (50.0)	93 (52.8)	
Intensive care unit	114 (37.0)	60 (45.5)	54 (30.7)	
Underlying disease				
Malignancy	87 (28.2)	42 (31.8)	45 (25.6)	0.281
Diabetes mellitus	52 (16.9)	24 (18.2)	28 (15.9)	0.709
Cardiovascular disease	50 (16.2)	26 (19.7)	24 (13.6)	0.204
Cerebrovascular disease	27 (8.8)	13 (9.8)	14 (8.0)	0.705
Liver cirrhosis	21 (6.8)	10 (7.6)	11 (6.3)	0.655
Chronic kidney disease	31 (10.1)	18 (13.6)	13 (7.4)	0.107
Charlson comorbidity index	4.0 [3.0–6.0]	5.0 [3.0–6.0]	4.0 [3.0–6.0]	0.208
SOFA score	5.0 [3.0–9.0]	7.0 [3.0–10.0]	4.0 [2.0–8.0]	0.001
Polymicrobial infection	66 (21.4)	22 (16.7)	44 (25.0)	0.104
Empirical antimicrobial treatment				
Piperacillin-tazobactam	92 (29.9)	40 (30.3)	52 (29.5)	0.986
Vancomycin	45 (14.6)	25 (18.9)	20 (11.4)	< 0.001
Teicoplanin	29 (9.4)	23 (17.4)	6 (3.4)	0.089
Linezolid	7 (2.3)	5 (3.8)	2 (1.1)	0.247
Definitive antimicrobial treatment				
Piperacillin-tazobactam	68 (22.1)	30 (22.7)	38 (21.6)	0.921
Vancomycin	59 (19.2)	20 (15.2)	39 (22.2)	0.161
Teicoplanin	65 (21.1)	24 (18.2)	41 (23.3)	0.343
Linezolid	46 (14.9)	39 (29.5)	7 (4.0)	< 0.001
Clinical outcome				
30-day mortality	96 (31.2)	48 (36.4)	48 (27.3)	0.114
60-day mortality	114 (37.0)	61 (46.2)	53 (30.1)	0.005
In-hospital mortality	131 (42.5)	72 (54.5)	59 (33.5)	< 0.001
Causative pathogen				
Resistant to				
Ampicillin	278 (90.3)	132 (100)	146 (83.0)	< 0.001
Ciprofloxacin	279 (90.6)	132 (100)	147 (83.5)	< 0.001
High-level gentamicin	73 (23.7)	52 (39.4)	21 (11.9)	< 0.001
High-level streptomycin	2 (0.6)	0 (0)	2 (1.1)	0.609
Tetracycline	37 (12.0)	11 (9.3)	26 (14.8)	0.123
Tigecycline	1 (0.3)	0 (0)	1 (0.6)	0.999
Quinupristin-dalfopristin	26 (8.4)	11 (8.3)	15 (8.5)	0.999
Vancomycin	128 (41.6)	128 (97.0)	0 (0)	< 0.001
Teicoplanin	107 (34.7)	107 (81.1)	0 (0)	< 0.001
Linezolid	0 (0)	0 (0)	0 (0)	–

### Antimicrobial resistance phenotypes of *E. faecium* blood isolates

Among the 308 *E. faecium* blood isolates, 132 (42.9%) showed positive results in *vanA* PCR, with none being positive in *vanB* PCR. All *vanA*-positive isolates were resistant to ampicillin and ciprofloxacin. High-level resistance to gentamicin was also identified in 39.4% (52/132) of the isolates, but none of them showed high-level resistance to streptomycin. Three-quarters of the isolates (75.0%, 99/132) exhibited VanA phenotypes, *i.e.*, high-level resistance to vancomycin (MIC, > 64 mg/L) and resistance to teicoplanin (MIC, ≥ 32 mg/L); 29 (22.0%) isolates exhibited VanD phenotypes, *i.e.*, high-level resistance to vancomycin (MIC, > 64 mg/L) and intermediate resistance (n = 22; MIC = 16 mg/L) or reduced susceptibility (n = 7; MIC = 8 mg/L) to teicoplanin. The remaining four (3.0%) isolates were susceptible to both vancomycin (MIC, 0.5–1 mg/L) and teicoplanin (MIC, 0.12–0.25 mg/L), indicating *vanA*-positive but vancomycin-susceptible (*vanA*^+^VS) phenotypes.

### Clinical outcome of patients with *E. faecium* BSI

Compared to those caused by *vanA*-negative *E. faecium,* BSIs caused by *vanA*-positive *E. faecium* occurred more frequently in inpatients (95.5% versus 85.2%; *P* value = 0.006) and in patients with a higher SOFA score (median value, 7.0 versus 4.0; *P* value = 0.001). The 30-day mortality rate was higher in patients with *vanA*-positive *E. faecium* BSI than in those with *vanA*-negative *E. faecium* BSI, but without statistical significance (36.4% versus 27.3%, *P* = 0.114). However, both the 60-day mortality (46.2% versus 30.1%; *P* value = 0.005) and in-hospital mortality (54.5% versus 33.5%; *P* value < 0.001) were significantly higher in *vanA*-positive *E. faecium* BSI patients than in *vanA*-negative *E. faecium* BSI patients (Figure S1).

### Genetic characteristics of *vanA*-positive *E. faecium* blood isolates

**Fig. 1:**
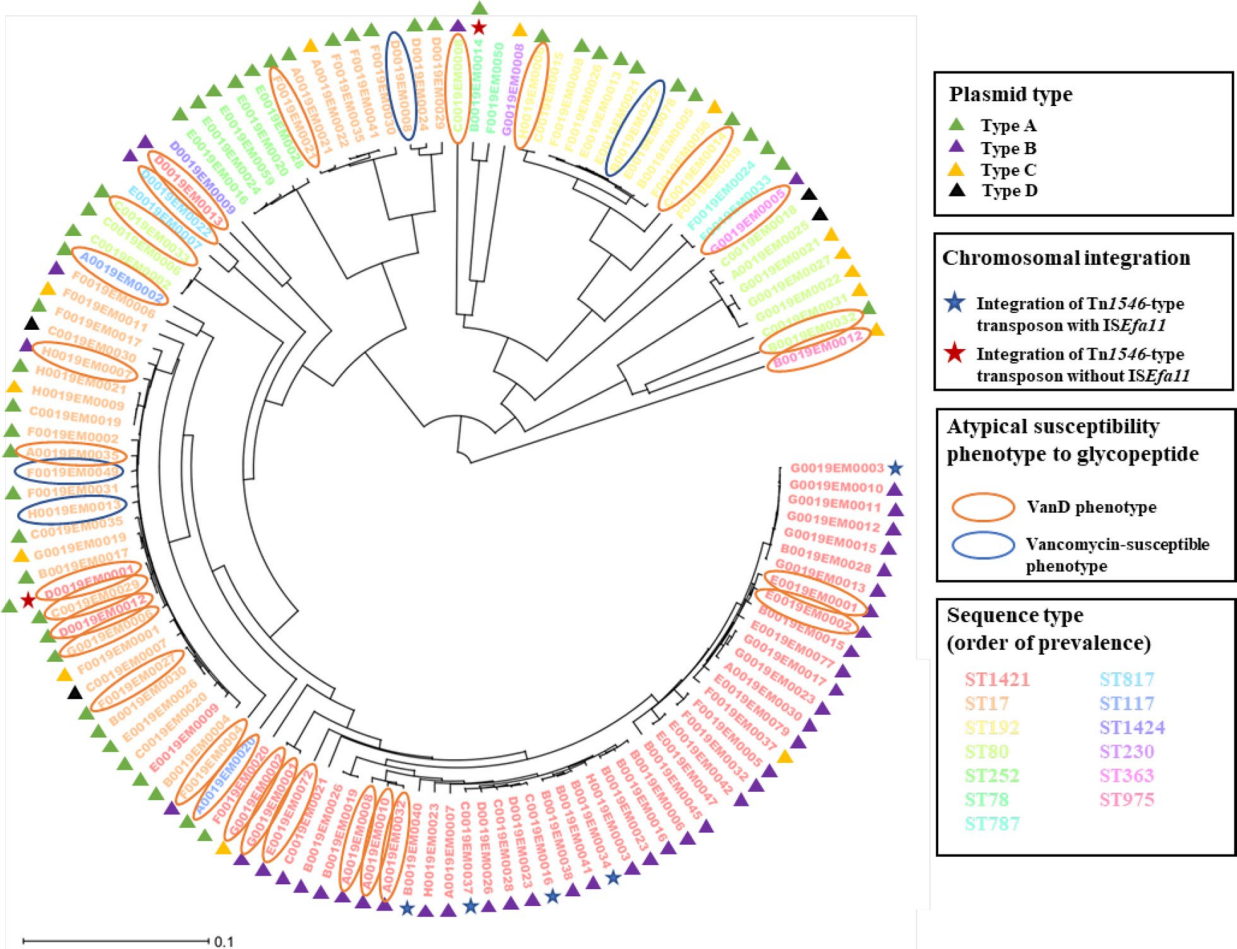
Phylogenetic tree based on cgMLST of *vanA*-positive *E. faecium* isolates

Circularized chromosomes were obtained from 120/132 *vanA*-positive *E. faecium* isolates by whole-genome sequencing, and mean value of coverage depth was 267.3 ranging from 92 to 620. Median size of circularized chromosomes was found to be 2,881,288 bp, ranging from 2,446,701 bp to 3,003,360 bp. The isolates carried one to three plasmids, with Rep A_N family and Inc18 family plasmids most frequently being identified. The most common strain type of *vanA*-positive blood isolate was ST1421 (n = 52), followed by ST17 (n = 36), ST80 (n = 15), ST192 (n = 12), and ST252 (n = 5) (Fig. 1). By cgMLST, 37 different complex types (CTs) were identified, and CT6141-ST17 (n = 23) was the most common, followed by CT6552-ST1421 (n = 20), CT6555-ST1421 (n = 15), and CT6554-ST192 (n = 10). One (n = 123) or two (n = 9) copies of the *vanA* operon were identified in each isolate, regardless of its location on a plasmid (n = 127) and/or the chromosome (n = 7); 2/132 isolates carried the *vanA* operon both on the chromosome and on a plasmid. Other resistance genes frequently identified on chromosomes were aminoglycoside-modifying enzyme-encoding genes *aac(6’)-Ii* (97.7%, n = 129) and *ant(9)-Ia* (60.6%, n = 80) and macrolide resistance-related genes *msr(c)* (97.7%, n = 129) and *erm(A)* (62.9%, n = 83).

### Structure of Tn*1546*-type transposons and glycopeptide resistance phenotypes

**Fig. 2:**
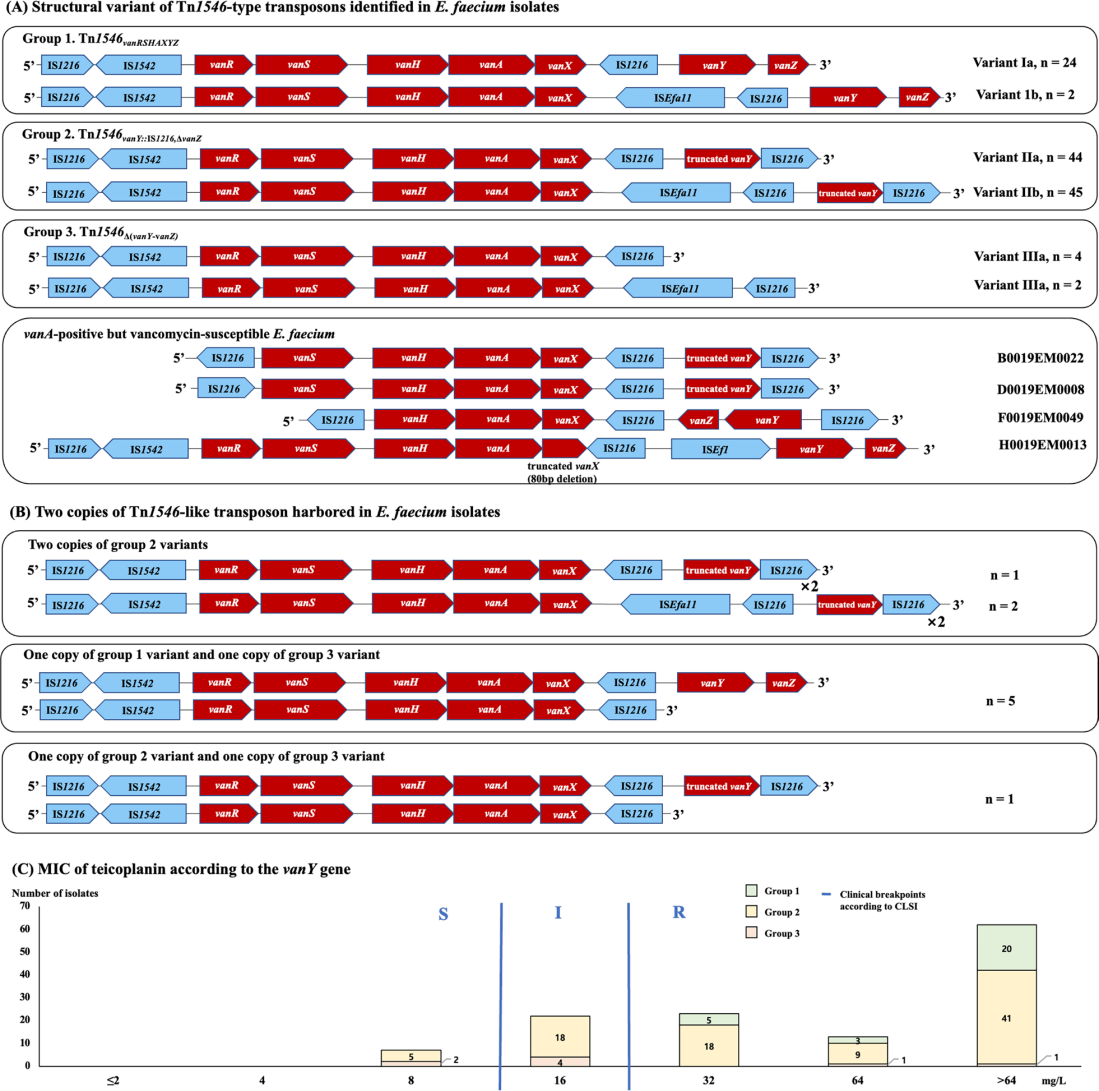
Structure of Tn*1546*-type transposons and teicoplanin MICs of *E. faecium* hosts according to the structure of *vanY*. Blue bars in the bar graph (C) indicate the clinical breakpoints according to the CLSI guideline, and red one indicates those according to the EUCAST

*vanA* operons were identified as a part of Tn*1546*-type transposons, and 128 blood isolates exhibiting a VanA or VanD phenotype carried one (n = 119) or two (n = 9) copies of the transposon classified as six structural variants both by deletion or truncation of *vanY* and *vanZ* and by insertion of IS*Efa11* between *vanX* and IS*1216* (Fig. 2A). The *vanS* gene of all 128 isolates showed nucleotide sequence variations resulting in three amino acid substitutions, L50V, E54Q, and Q69H, compared with the *vanS* gene of pIP501 [7, 30]. The remaining four isolates with *vanA*^+^VS phenotypes were found to carry a structurally impaired *vanA* operon, either lacking one or both regulatory genes *vanR* and *vanS* or having a truncated D-alanyl-D-alanine dipeptidase gene *vanX*, on a plasmid.

The 128 isolates with the VanA or VanD phenotype were grouped according to the *vanA* operon copy number and structure. Group 1 isolates (n = 24) showing the typical VanA phenotype carried a plasmid harboring a copy of a Tn*1546*-type transposon (Tn*1546*_*vanRSHAXYZ*_) with all seven component genes of the *vanA* operon, with (variant Ib, n = 2) or without (variant Ia, n = 22) insertion of IS*Efa11*. Group 2 isolates (n = 89) carried a copy of the Tn*1546* variant with both truncation of *vanY* and deletion of *vanZ* by insertion of an IS*1216* into *vanY* (Tn*1546*_*vanY*::IS*1216*,Δ*vanZ*_) with (variant IIb, n = 45) or without (variant IIa, n = 44) insertion of IS*Efa11*. The isolates exhibited VanA or VanD phenotype, and the Tn*1546*_*vanY*::IS*1216*,Δ*vanZ*_ variants were found to be located either on a plasmid (n = 85) or on the chromosome (n = 4). The length of remnant *vanY* in Tn*1546*_*vanY*::IS*1216*,Δ*vanZ*_ varied from 165 to 901 bp according to the insertion site, which did not show any correlation with teicoplanin MICs. Group 3 isolates (n = 6) exhibiting the VanD phenotype carried a copy of the Tn*1546*-type transposon with deletion of both *vanY* and *vanZ* [Tn*1546*_Δ(*vanY-vanZ)*_] with (variant IIIb, n = 2) or without (variant IIIa, n = 4) insertion of IS*Efa11* on a plasmid. Nine isolates harbored two copies of Tn*1546*-type transposons on a plasmid and/or on the chromosome (Fig. 2B). Of them, five and one isolates exhibiting the VanA phenotype possessed one each copy of Tn*1546*_*vanRSHAXYZ*_ and Tn*1546*_Δ(*vanY-vanZ)*_ and Tn*1546*_*vanY*::IS*1216*,Δ*vanZ*_ and Tn*1546*_Δ(*vanY-vanZ)*_, respectively. The remaining three isolates carried two copies of Tn*1546*_*vanY*::IS*1216*,Δ*vanZ*_ and showed the VanA (n = 2) or VanD (n = 1) phenotype (Fig. 2B).

### Chromosomal *vanA* operon

**Fig. 3:**
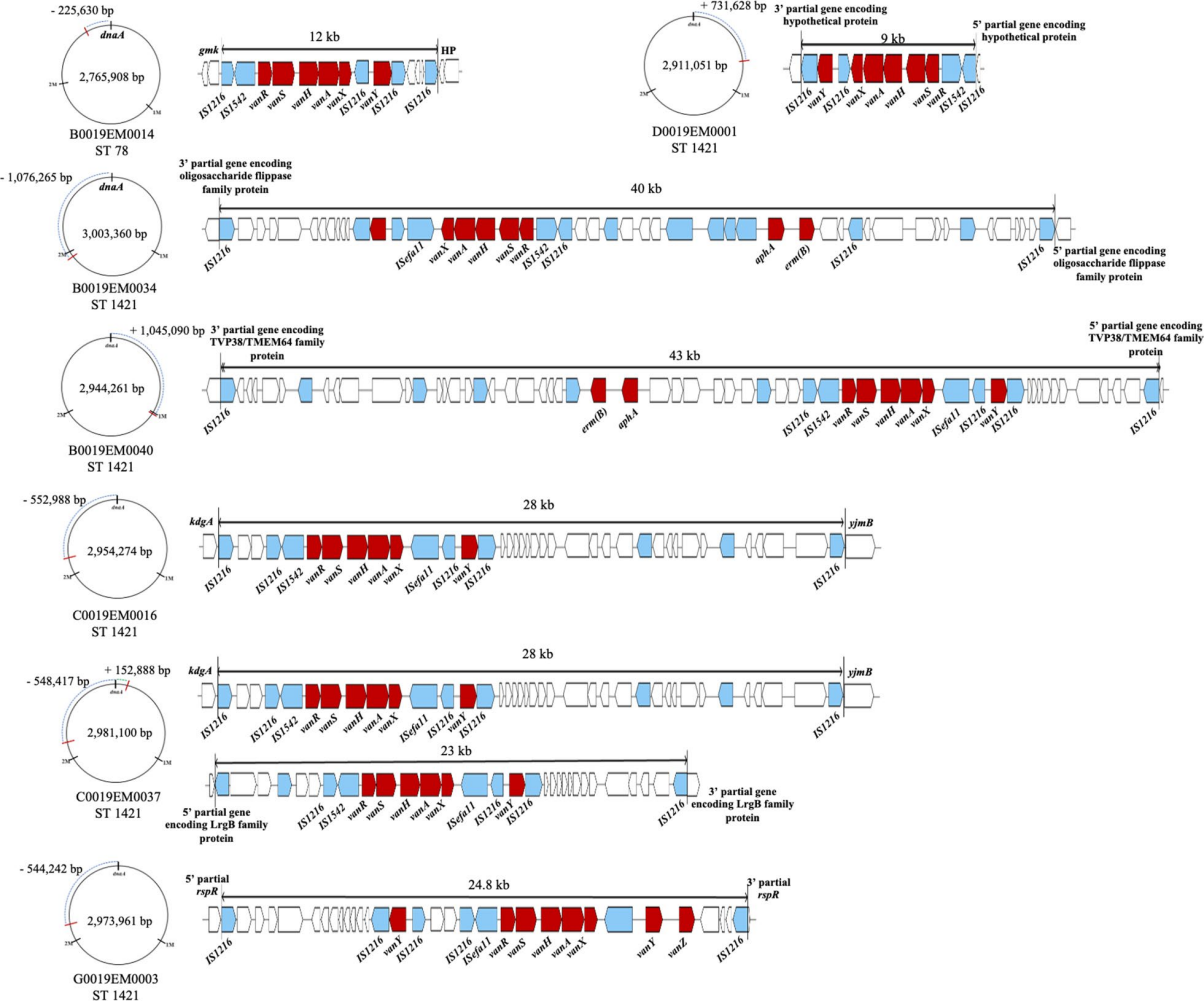
Structure and location of the chromosomal *vanA* operon. The red bars in chromosome indicate the insertion sites of Tn*1546*-type transposon. Red arrows indicate the genes identified as resistance determinants, and blue arrows indicate the insertion sequences

One (n = 6) or two (n = 1) copies of the *vanA* operon were identified on the chromosome in seven isolates of CT6555-ST1421 (n = 4), CT6141-ST1421 (n = 1), CT6552-ST1421 (n = 1), and CT6557-ST78) (n = 1), and two of them carried an additional *vanA* operon-harboring plasmid (Fig. 3). The *vanA* operons found on the chromosome were always identified in an insertion unit with (n = 6) or without (n = 2) plasmid-originated components, resulting in variable sizes of the units, ranging from 9 to 43 kb. All eight insertion units were shown to be flanked by a pair of IS*1216* of the same or opposite orientations and left (5*'*-GGT TCT GTT GCA AAG TTT TAA ATC TAC TAT CAA ATA AGG TAG AAT AG-3*'*) and right (5*'*- GGT TCT GTT GCA AAG TTT TAA ATA AAG AAT AAA ATC CTT ACG GTA TCT AT-3*'*) inverted repeats. The insertion events on the chromosome were shown to be neither site-specific nor nucleotide sequence-specific, occurring in coding regions (n = 5) or in intergenic regions (n = 3). Of note, both isolates C0019EM0016 and C0019EM0037 of CT6555-ST1421 recovered in a hospital with a 7-month gap shared an insertion unit at the same location on the chromosome, indicating a clone; however, the isolate C0019EM0037 carried another insertion unit at a different location on the chromosome, suggesting that another independent insertion event occurred.

### VR-plasmids harboring the *vanA* operon

A total of 127 VR-plasmids carrying one (n = 122) or two (n = 5) copies of Tn*1546*-type transposons were identified. Circular plasmids of the Inc18 family (n = 96) were most common, followed by putative linear plasmids of the RepB family (n = 21); a circular plasmid of hybrid Inc18:RepA_N was also identified. The remaining nine plasmids were nontypeable due to failure in plasmid circularization (n = 5) or in identification (n = 4) of the plasmid replication origin.

**Fig. 4:**
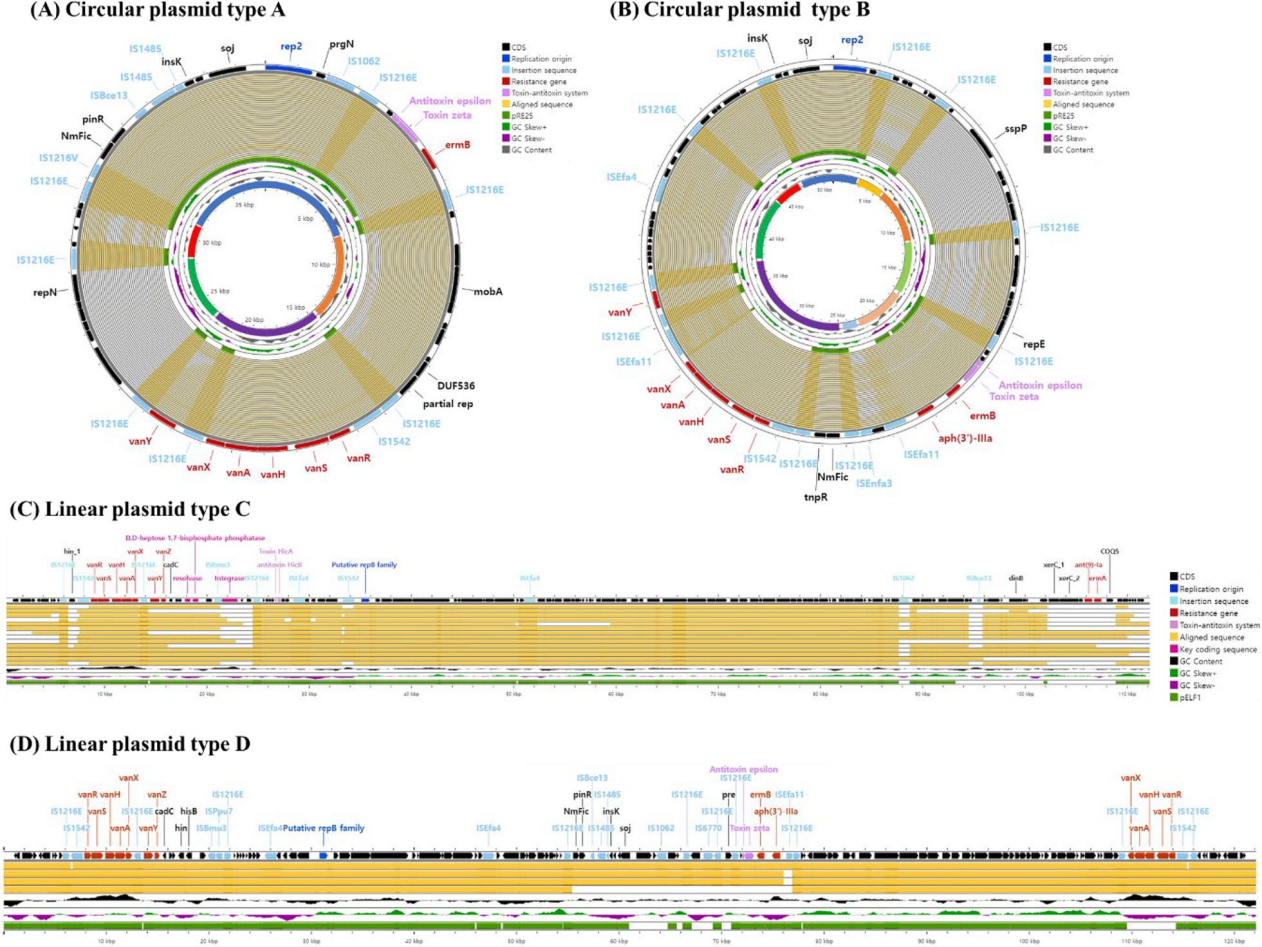
Structure of four types of plasmids containing Tn*1546*-type transposons

All circular plasmids of the Inc18 family were found to share 14–15 kb-sized derivatives from the plasmid pRE25, including a *rep2* replication origin, the *erm(B)* gene, and a zeta-epsilon toxin-antitoxin system, but to lack the probable conjugation regions (ORF25 to ORF39 of pRE25) [31], compatible with the unsuccessful results in conjugation experiments for all *E. faecium* isolates carrying the plasmids. The 96 circular plasmids were divided into types A (n = 50) and B (n = 46) according to both sequence similarity (> 60%) and Tn*1546* variant type (Fig. 4A and B). Type A plasmids had a median size of 32,082 bp, ranging from 18,701 bp to 43,334 bp, and were most frequently identified in *E. faecium* isolates of ST17 (46%, 23/50). Type A plasmids possessed either Tn*1546* variant IIa (n = 45) or variant IIIa (n = 3), except for two plasmids harboring a Tn*1546*-type transposon with a structurally impaired *vanA* operon, both identified in *vanA*^+^VS isolates (Table 2). Type B plasmids had a median size of 42,610 bp, ranging from 27,214 bp to 51,084 bp, and were mostly (80.4%, 37/46) identified in *E. faecium* isolates of ST1421. Tn*1546* variants identified in type B plasmids always showed insertion of IS*Efa11* between *vanX* and IS*1216*, and variant IIb (n = 41) was most common, followed by variant Ib (n = 3) and variant IIIb (n = 2). Most (97.8%, 45/46) type B plasmids also had an aminoglycoside resistance determinant *aph(3')-IIIa.*

**Table 2:** Characteristics of plasmids harboring the *vanA* operon

	pRE25-like type A (n = 50)	pRE25-like type B (n = 46)	pELF1-like type C (n = 16)	pELF1-like Type D (n = 5)
Schematic appearance	Circular plasmid	Circular plasmid	Putative linear plasmid with one hairpin end	Putative linear plasmid with both hairpin ends
Rep family (incompatibility type)	rep2 (Inc18)	rep2 (Inc18)	putative RepB family	putative RepB family
Size, median (range)	32,082 (18,701–43,334)	42,610 (27,214–51,084)	106,938 (72,306–112,181)	118,791 (105,273–150,375)
Toxin-antitoxin system	zeta-epsilon	zeta-epsilon	hicA-hicB	zeta-epsilon, hicA-hicB
Coresistance gene				
* erm(A)*	0 (0)	0 (0)	5 (33.3)	0 (0)
* erm(B)*	50 (100)	46 (100)	0 (0)	4 (80.0)
* aph(3')-III*	0 (0)	46 (100)	0 (0)	4 (80.0)
* ant(9)-Ia*	0 (0)	0 (0)	6 (40.0)	0 (0)
Strain type				
ST1421	4 (8.0)	37 (80.5)	2 (0)	0 (0)
ST17	23 (46.0)	5 (10.9)	5 (33.3)	2 (40.0)
ST192	9 (18.0)	0 (0)	1 (6.7)	0 (0)
ST80	4 (8.0)	1 (2.2)	5 (33.3)	3 (60.0)
ST252	5 (10.0)	0 (0)	0 (0)	0 (0)
Others	5 (10.0)	3 (6.6)	2 (13.3)	0 (0)
Structure of Tn*1546*-type transposon				
Tn*1546*_*vanRSHAXYZ*_	0 (0)	3 (6.6)	14 (87.4)	5 (50.0)
Tn1546_*vanY*::IS*1216*,Δ*vanZ*_	45 (90.0)	41 (87.0)	0 (0)	0 (0)
Tn*1546*_Δ(*vanY-vanZ*)_	3 (6.0)	2 (6.6)	1 (6.3)	5 (50.0)
Structurally impaired *vanA* operon	2 (4.0)	0 (0)	1 (6.3)	0 (0)
Vancomcyin-reistsant phenotype				
VanA phenotype	42 (84.0)	38 (82.7)	14 (87.4)	5 (100)
VanD phenotype	6 (12.0)	8 (17.4)	1 (6.3)	0 (0)
VS phenotype	2 (4.0)	0 (0)	1 (6.3)	
Copy number of *vanA* operon				
1 copy	50 (100)	45 (97.8)	16 (100)	0 (0)
2 copies	0 (0)	1 (2.2)	0 (0)	5 (100)
Conjugation ability	0 (0)	0 (0)	8 (66.7)	2 (20.0)
Conjugation frequency				
Per number of donor cells, median (range)	–	–	3.39 × 10^–6^ (5.75 × 10^–7^–1.18 × 10^–5^)	1.91 × 10^–6^ (1.75 × 10^–6^–2.07 × 10^–6^)
Per number of recipient cells, median (range)	–	–	9.38 × 10^–8^ (6.00 × 10^–10^–3.87 × 10^–7^)	7.40 × 10^–9^ (5.05 × 10^–9^–9.76 × 10^–9^)

A putative replication origin of the RepB family was found in 21 putative linear plasmids with either one (type C, n = 16) or two (type D, n = 5) hairpin ends composed of inverted tandem repeat sequences of 2 kb with 5*'*-TATA-3*'* hairpin loops (Fig. 4C and D). The plasmids exhibited homology of approximately 70% with the linear plasmid pELF1 [32], and they shared putative transfer-related components *ftsK* and *parA*. More than half (57.1%, 12/21, 10 type C and two type D) of the plasmids were successfully conjugated to the recipient *E. faecium* DSM13589. The type C plasmids had a median size of 106,938 bp, ranging from 72,306 to 112,181 bp. The single hairpin end of the plasmids were observed to harbor a copy of Tn*1546*_*vanRSHAXYZ*_ (14/16), Tn*1546*_Δ(*vanY-vanZ)*_ (n = 1), or a Tn*1546*-type transposon with a structurally impaired *vanA* operon (n = 1); other resistance determinants *ant(9)-Ia* (n = 6) and *erm(A)* (n = 5) were also identified at the opposite end of the plasmids. The type D plasmids had a median size of 118,791 bp, ranging from 102,459 to 161,239 bp, and harbored two copies of the Tn*1546*-type transposon, each copy of Tn*1546*_*vanRSHAXYZ*_ and Tn*1546*_Δ(*vanY-vanZ)*_ at each hairpin end. The plasmids also harbored the resistance determinants *erm(B)* and *aph(3')-IIIa*.

### Bacterial growth rate

**Fig. 5:**
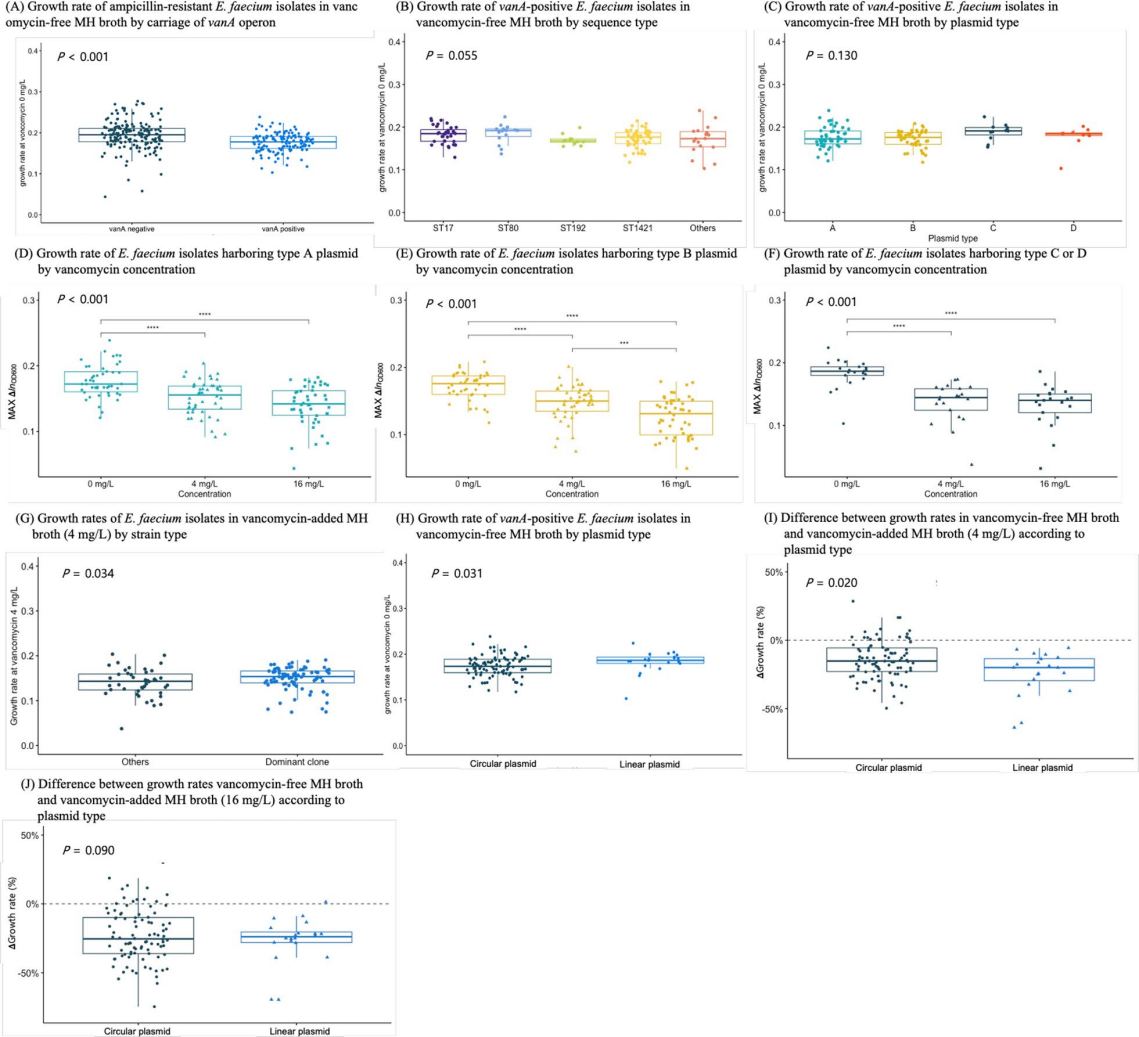
Bacterial growth rates of *E. faecium* blood isolates. *** indicate *P* value < 0.001

The median growth rate [Max(∆*ln*OD_600_/s)] of *vanA*-negative ampicillin-resistant *E. faecium* blood isolates in MH broth was 0.195 (1st to 3rd interquartile range, 0.178 to 0.211), which was significantly higher than that of *vanA*-positive ampicillin-resistant isolates (median value, 0.178; 1st to 3rd interquartile range, 0.162 to 0.191; *P* < 0.001) (Fig. 5A).

The growth rates of four *vanA*^+^VS isolates (median value, 0.200; range, 0.156 to 0.220) were similar to those of *vanA*-negative isolates. Growth rates of *vanA*-positive isolates did not differ by strain type or R plasmid type carrying the *vanA* operon (Fig. 5B and C). Addition of vancomycin at concentrations of 4 mg/L and 16 mg/L to MH broth resulted in a significant decrease in the growth rates of *vanA*-positive *E. faecium* isolates, regardless of plasmid type (Fig. 5D–F). Notably, the growth rates of ST1421 and ST17 *vanA*-positive *E. faecium* isolates at a vancomycin concentration of 4 mg/L (median value, 0.154; 1st to 3rd interquartile range, 0.139–0.166) were significantly faster than those of *vanA*-positive isolates of other STs (median value, 0.143; 1st to 3rd interquartile range, 0.122–0.160; Fig. 5G). The difference between growth rates in MH broth without vancomycin and with vancomycin at a concentration of 4 mg/L was significantly lower for isolates carrying a type A or B circular plasmid than those carrying a type C or D linear plasmid (Fig. 5H; *P* = 0.020).

## Discussion

Our experiments revealed a significant difference in growth rates between *vanA*-positive and *vanA*-negative *E. faecium* blood isolates in MH broth, suggesting that it is costly to bacterial hosts in antimicrobial-free environments to carry the *vanA* operon, regardless of the location of the operon on a plasmid or the chromosome. This fitness burden might be attributed to the basal expression level of the *vanA* gene even in antimicrobial-free environments [33]. The growth rates of four *vanA*^+^VS isolates with a structurally impaired *vanA* operon were similar to those of *vanA*-negative isolates in our study, though a statistically significant difference with those of VREfm isolates was not observed due to the small number of cases. Complete inactivation of the *vanA* operon in *vanA*^+^VS isolates might be a way to overcome the fitness burden for *vanA*-carrying *E. faecium* bacterial hosts when exposed to antimicrobial-free environments [34]. These findings provide evidence for the importance of reducing vancomycin pressure through antimicrobial stewardship in clinical fields to prevent dissemination of VREfm.

Addition of vancomycin at a sub-MIC concentration of 4 mg/L to MH broth significantly slowed the growth rates of VREfm isolates compared with those in MH broth without antimicrobials. Both increased fitness burden to bacterial hosts by increased expression level of the *vanA* gene and growth inhibition effects of vancomycin might affect the growth rates of VREfm isolates [35]. It is noteworthy that the difference in growth rate was significantly larger in isolates carrying a type C or D putative linear conjugative plasmid (approximately 100–200 kb) than in those carrying a type A or B circular nonconjugative plasmid (approximately 30–40 kb), indicating that the former plasmids are costlier to bacterial hosts under vancomycin pressure. Furthermore, both predominant VREfm clones of ST1421 and ST17, which mostly harbored a type A or B plasmid, exhibited significantly faster growth rates in MH broth with vancomycin than other VREfm isolates. These findings evidence the reason for the success of predominant VREfm clones in hospital settings under persistent vancomycin pressure. However, significant difference was not identified in the subgroup analyses with 32 ST17 strains including 26 with circular plasmids and 6 with putative plasmids due to the limited number of strains belonging to same ST. Further investigation about fitness cost according to the bacterial hosts and their plasmid type should be performed.

Bacterial hosts might possess a nonconjugative plasmid harboring the Tn*1546*-type transposon in two possible ways: (1) loss of essential components for conjugation on the plasmid by genetic recombination events after acquisition of the plasmid by conjugation [36, 37] and (2) intracellular mobilization of the Tn*1546*-type transposon from a conjugative plasmid to a nonconjugative plasmid [38]. In our study, most of the type A and B nonconjugative plasmids harbored Tn*1546*_*vanY*::IS*1216*,Δ*vanZ*_, whereas most of the type C and D conjugative plasmids harbored Tn*1546*_*vanRSHAXYZ*_. Tn*1546*-type transposons were always found to be bracketed by IS*1216* elements, and both truncation of *vanY* and deletion of *vanZ* in types A and B plasmids might result from insertion of IS*1216* at the random sequences of *vanY* of Tn*1546*_*vanRSHAXYZ*_, leaving variable sizes of *vanY* remnants. Furthermore, the EFM isolate G0019EM0008 co-carried a type C plasmid harboring Tn*1546*_*vanRSHAXYZ*_ with an additional pRE25-like plasmid, sharing a backbone structure with type A plasmids but lacking Tn*1546*, which might constitute a snapshot before an intracellular mobilization event of the transposon.

Tn*1546*-type transposons were also identified on the chromosomes of seven VREfm isolates with or without surrounding components of type B circular plasmids, suggesting the origin of the transposons. Insertion of plasmid-originated antimicrobial resistance determinants into the chromosome has also been reported for other bacterial species, such as IS*Ecp1-bla*_CTX-M_ in *Escherichia coli* and *Salmonella* species, and IS*Aba1-bla*_OXA-23_ in *Acinetobacter baumannii* [39–41]. This phenomenon might indicate an internalization process of antimicrobial resistance determinants by bacterial hosts in response to constant antimicrobial pressure. Notably, most VREfm isolates harboring the Tn*1546*-type transposon on their chromosome were shown to belong to ST1421. The ST1421 VREfm isolates, which were first identified in Australia, had a large chromosomal inversion resulting in deletion of 3.5- to 8.7-kb chromosomal sequences, including the *pstS* gene [42]. The high genome plasticity of this notorious clone might be a preferred condition for insertion of the Tn*1546*-type transposons on the chromosome.

Tn*1546*_*vanY*::IS*1216*,Δ*vanZ*_ was found to confer variable levels of resistance against teicoplanin to bacterial hosts, from reduced susceptibility (MIC = 8 mg/L) to high-level resistance (MIC ≥ 32 mg/L), but Tn*1546*_*vanRSHAXYZ*_ conferred high-level resistance, consistent with previous reports [43, 44]. However, identical nucleotide sequence variations in *vanS* were identified in all VREfm isolates regardless of their susceptibility phenotype to teicoplanin, inconsistent with a previous report [30], indicating that they might not cause functional changes in VanS protein capability.

A limitation of this study is that the VREfm isolates were collected in a single country, South Korea, and the local distribution of the strain type of VREfm isolates, type of plasmids carrying the Tn*1546*-type transposon, and structure of the Tn*1546*-type transposon might be reflected in the results of this study. Another limitation is that the growth rate of *E. faecium* blood isolates were determined at nutrient-rich conditions, therefore, further investigation including competitive growth and in vivo adaptation experiments and should be performed to clarify the effects of fitness costs.

## Conclusions

The possession of Tn*1546*-type transposon harboring *vanA* operon was costly to bacterial hosts in antimicrobial-free environment, which provide evidence for the importance of reducing vancomycin pressure for prevention of VREfm dissemination through antimicrobial stewardship in clinical fields. Fitness burden to bacterial hosts of Tn*1546*-type transposon was varied by type and size of the *vanA* operon-harboring plasmid, which could have an impact on successful dissemination of the epidemic clones.

### Supplementary Information

Additional file 1.

## Data Availability

The genome data of this study are available from National Centers for Bio Informatics in BioProject under accession PRJNA983092.

## Abbreviations

VREfm: Vancomycin-resistant *Enterococcus faecium*
VR-plasmid: Plasmid with resistance determinants against vancomycin
BSI: Bloodstream infection
SOFA: Sepsis-related Organ Failure Assessment
MIC: Minimum inhibitory concentration
MLST: Multilocus sequence typing
cgMLST: Core genome multilocus sequence typing
MH: Mueller–Hinton
OD_600_: Optical density at 600 nm
