# Supplementary material for: Fitness costs of Tn1546-type transposons harboring the vanA operon by plasmid type and structural diversity in Enterococcus faecium
Source: Ann Clin Microbiol Antimicrob. 2024 Jul 8;23:62. doi: 10.1186/s12941-024-00722-2 (PMC11229256; doi:10.1186/s12941-024-00722-2)
Supplement: Supplementary file 1 — Additional file 1. [file 12941_2024_722_MOESM1_ESM.docx]

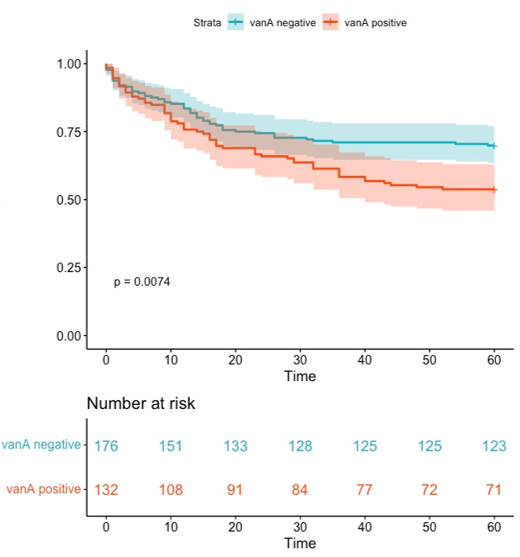


Figure S1. Survival curve of patients with E. faecium bloodstream infection. Kaplan-Meier curve was constructed, and log-rank test was performed to figure out statistical significance.

Table S1. Characteristics of *E. faecium* isolates having *vanA* operon in their chromosome

| Case | Size of chromosome (bp) | Copy number | MLST | insertion site location from *dnaA* | site of insertion fragment | Insertion site | Structure of insertion site |
| --- | --- | --- | --- | --- | --- | --- | --- |
| B0019EM0014 | 2,765,908 | 1 | ST78 | -225,630 | 12kb | Intergenic region | Intergenic region between *gmk* and hypothetical protein |
| B0019EM0034 | 3,003,360 | 1 | ST1421 | -1,076,265 | 40kb | Reading frame | Gene encoding oligosaccharide flippase family protein |
| B0019EM0040 | 2,944,261 | 1 | ST1421 | 1,045,090 | 43kb | Reading frame | Gene encoding TVP38/TMEM64 family protein |
| C0019EM0016 | 2,954,274 | 1 | ST1421 | -552,988 | 28kb | Reading frame | Intergenic regions between *yjmB* and *kdgG* |
| C0019EM0037 | 2,981,100 | 2 | ST1421 | 152,888  -548,417 | 23kb  28kb | Intergenic region  Reading frame | Gene encoding LrgB family protein  Intergenic regions between *yjmB* and *kdgG* |
| D0019EM0001 | 2,911,051 | 1 | ST1421 | 731,628 | 9kb | Reading frame | Gene encoding hypothetical protein |
| G0019EM0003 | 2,973,961 | 1 | ST1421 | -544,242 | 41kb | Reading frame | *rspR* |
